# Coevolutionary interplay: Helminths-trained immunity and its impact on the rise of inflammatory diseases

**DOI:** 10.7554/eLife.105393

**Published:** 2025-04-15

**Authors:** Eugenio Antonio Carrera Silva, Juliana Puyssegur, Andrea Emilse Errasti

**Affiliations:** 1 EACS and JP Institute of Experimental Medicine, National Scientific and Technical Research Council, National Academy of Medicine (IMEX-CONICET-ANM) Buenos Aires Argentina; 2 AEE Institute of Pharmacology, School of Medicine, University of Buenos Aires Buenos Aires Argentina; 3 National Scientific and Technical Research Council (CONICET) Buenos Aires Argentina; Murdoch Children's Research Institute Australia; Indian Institute of Science Education and Research (IISER) India

**Keywords:** trained immunity, helminths, extracelular vesicles, EVs, excretory/secretory products, ESPs, gut biome, inflammatory disorders

## Abstract

The gut biome, a complex ecosystem of micro- and macro-organisms, plays a crucial role in human health. A disruption in this evolutive balance, particularly during early life, can lead to immune dysregulation and inflammatory disorders. ‘Biome repletion’ has emerged as a potential therapeutic approach, introducing live microbes or helminth-derived products to restore immune balance. While helminth therapy has shown some promise, significant challenges remain in optimizing clinical trials. Factors such as patient genetics, disease status, helminth species, and the optimal timing and dosage of their products or metabolites must be carefully considered to train the immune system effectively. We aim to discuss how helminths and their products induce trained immunity as prospective to treat inflammatory and autoimmune diseases. The molecular repertoire of helminth excretory/secretory products (ESPs), which includes proteins, peptides, lipids, and RNA-carrying extracellular vesicles (EVs), underscores their potential to modulate innate immune cells and hematopoietic stem cell precursors. Mimicking natural delivery mechanisms like synthetic exosomes could revolutionize EV-based therapies and optimizing production and delivery of ESP will be crucial for their translation into clinical applications. By deciphering and harnessing helminth-derived products’ diverse modes of action, we can unleash their full therapeutic potential and pave the way for innovative treatments.

## Trained immunity

Trained immunity describes the long-term functional reprogramming of innate host defense mechanisms or a de facto innate immune memory, where environmental stimuli, such as the commensal host biome, their products, or even endogenous molecules, can alter a second immune response by metabolic or epigenetic reprogramming (Netea et al., 2020).

Immunological memory has been traditionally associated with the adaptive arm of the immune response in vertebrates. However, unlike vertebrates, most species rely exclusively on innate immunity for host defense. Therefore, a critical evolutionary attribute like immunological memory is unlikely to be limited to adaptive immunity and absent in the innate response in the whole spectrum of living organisms (Divangahi et al., 2021).

In the last decades, this understanding that only adaptive immunity can shape immunological memory has been increasingly challenged. After facing a primary stimulus, epigenetic and metabolic alterations of bone marrow progenitor cells and functional changes of tissue immune cell populations result in enhanced immune responses against a secondary encounter. This long-term reprogramming process has been named ‘innate immune memory’ or ‘trained immunity’ (Netea et al., 2011), adding new complexity to our previous knowledge of immunological memory, an attribute limited to antigen-specific responses of the adaptive compartment of the immune system (Netea et al., 2016).

The specificity of adaptive immune memory in vertebrates is ensured through immunoglobulin gene recombination and clonal expansion, while nonspecific trained innate immune response relies on still not fully understood mechanisms closely intertwined epigenetic, transcriptional, and metabolic pathways (Vuscan et al., 2024).

In animals, evidence comes from studies showing that vaccination with the tuberculosis vaccine Bacillus Calmette-Guérin (BCG) - the most commonly used vaccine worldwide - induces T cell-independent protection against secondary infections with *Candida albicans, Schistosoma mansoni*, or influenza (Spencer et al., 1977; Tribouley et al., 1978; van ’t Wout et al., 1992). In human volunteers, BCG vaccination protects against an experimental infection with the yellow fever vaccine virus (Arts et al., 2018). Moreover, extensive epidemiological studies have reported protective heterologous effects of both BCG and measles vaccination (Arts et al., 2018; Benn et al., 2013; Goodridge et al., 2016).

In addition, herpesvirus latency increases resistance to the bacterial pathogens *Listeria monocytogenes* and *Yersinia pestis* (Barton et al., 2007), with protection achieved through enhanced production of IFNγ and systemic activation of macrophages. Similarly, infection with the helminthic parasite *Nippostrongylus brasiliensis* induces a long-term macrophage phenotype that damages the parasite and induces protection from reinfection independently of T and B lymphocytes (Chen et al., 2014).

It is also significant to emphasize that while trained immunity is an evolutionary trait that increases fitness against pathogenic microbes, it may also be maladaptive in the context of chronic inflammatory diseases. Thus, trained immunity may conversely contribute to the pathophysiology of cardiovascular, autoinflammatory, and neurodegenerative diseases. Indeed, in addition to microbial exposure and products, endogenous molecules can induce trained immunity. For instance, a Western-type diet prompts trained immunity in animal models of atherosclerosis, which perseveres even after a switch to a healthy chow diet (Christ et al., 2018).

In addition, it is notable that trained immunity induced by BCG vaccination was associated in the enhanced induction of immune self-tolerance in models of autoimmunity such as type 1 diabetes and multiple sclerosis (Ristori et al., 2018). At least in some autoimmune conditions, the advantageous effects of BCG vaccination, denote an interesting aspect of the host-microbe interaction in the pathophysiology and treatment of immune-mediated diseases. Although the precise molecular mechanisms are not entirely understood, this data supports the idea that exposure to microbes may relieve conditions characterized by chronic inflammation and injury of tissues, such as inflammatory and autoimmune diseases.

## The gut biome and chronic inflammatory diseases

The gut biome is the ecosystem composed of bacteria, viruses, fungi, archaea, and helminths that inhabit our gastrointestinal tract. After millennia of coevolution, humans have established a symbiotic relationship with their commensal biome, in particular, as an essential contributor to the development and training of the host immune system (Belkaid and Hand, 2014; Ley et al., 2006; Zheng et al., 2020). Dysbiosis, defined as an imbalance in the microbiome composition or function due to loss of beneficial microorganisms, overgrowth of pathogens or loss of overall diversity, has gained attention in recent years, as evidence suggests it could play a crucial role in the development of inflammatory diseases (Petersen and Round, 2014; Hand et al., 2016; Alagiakrishnan et al., 2024).

Research suggests that a disturbance in the interaction between host microbes early in life could leave an imprint on the immune system, leading to excessive reactivity later on, predisposing the individual to inflammatory disorders (Al Nabhani and Eberl, 2020). Furthermore, studies demonstrate that the mode of delivery significantly impacts microbiome composition during the first years of life; for example, a caesarian section leads to delayed colonization by the phylum Bacteroidetes, which is associated with reduced T helper 1 cells (Th1) responses during infancy and a higher incidence of allergies (Jakobsson et al., 2014). In adulthood, the microbiome becomes relatively stable, and it is generally considered healthy when characterized by high diversity (large number of species) and high evenness (high abundance of numerous species) (Bradley and Haran, 2024; Odamaki et al., 2016; Jeffery et al., 2016). The reason appears to be that a more diverse microbiome covers a more significant number of functional niches, with different species supporting different aspects of the host’s health and homeostasis, including nutrition, protection against pathogens, immune system regulation, maintenance of gut barrier integrity, xenobiotic and drug metabolism, among others (Sommer et al., 2017; Shepherd et al., 2018; Gibbons, 2019).

The last century has witnessed a rise in allergies and inflammatory and autoimmune diseases, with a higher prevalence in industrialized societies. This was also supported by the observation that people migrating from an area with a low incidence of immune-mediated diseases to an area with a higher one increases the likelihood of acquiring such disorders (Bach, 2002; Alter et al., 1978; Bodansky et al., 1992; Hammond, 2000). The first postulate to explain these observations was the ‘hygiene hypothesis’, coined in 1988 by David Barker and supported later by David Strachan in 1989 (Barker et al., 1988; Strachan, 1989; Perkin and Strachan, 2022). Nonetheless, a more comprehensive and evolutionary explanation emerged in 2003 with the ‘old friends hypothesis’, proposed by Graham A. W. Rook, which states that a lack of exposure to certain host evolved-dependent organisms that stimulate immunoregulatory pathways results in an overreactive immune system that targets autoantigens and harmless allergens (Yazdanbakhsh et al., 2002; Rook, 2023; Rook et al., 2004; Scudellari, 2017).

Helminths play a central role in the ‘old friends hypothesis’ because, until the onset of industrialization, our immune system had to evolve alongside the presence of chronic parasitic infections within the host, as happens in wild animals. The depletion of helminths and other microorganisms from our biome (see Box 1), coupled with different environmental factors like reduced vitamin D levels and increased psychological stress, has potentially led to an ‘evolutionary mismatch,’ a condition in which an organism becomes maladapted to a rapidly changing environment. (Parker and Ollerton, 2013; McSorley and Maizels, 2012; Panelli et al., 2020).

Box 1. The **Biome Depletion Theory** states that the loss of species diversity from the human body’s ecosystem in modern industrialized countries leads to immune dysregulation and a subsequent increase in the prevalence of chronic inflammatory-associated diseases. This paradigm appreciates the importance of an array of microbes and helminths as essential for immune system development and regulation (Parker and Ollerton, 2013, von Hertzen et al., 2011).

The **Gut-Brain Axis** proposed the gut microbiota as a central environmental contributor to immune-mediated diseases such as multiple sclerosis (MS) (Correale et al., 2022).

**Figure 1.**
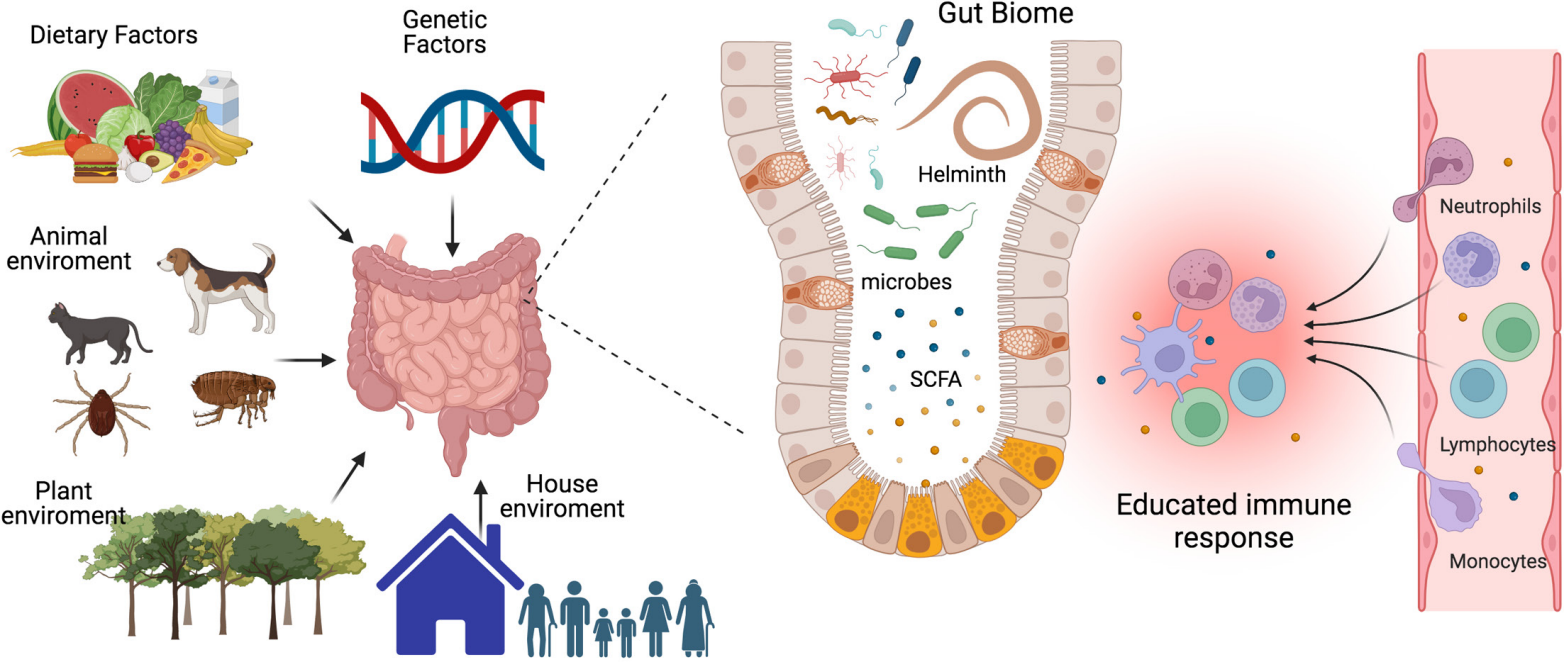
The interplay between gut microbiota, immune system, and environmental factors. Our immune system’s development, maturation, and response to challenges are significantly influenced by the composition of our gut biome. This complex ecosystem is shaped not only by host genetics but also by critical environmental factors such as diet, lifestyle, and exposure to animals, plants, and other individuals. The micro- and macro-biota play a vital role in maintaining both gut and systemic immune homeostasis by modulating both innate and adaptive immune responses. Helminths can directly or through the microbiota or metabolic changes educate or train our immune system to promote regulatory networks that benefit from a less aggressive inflammatory response. A deeper understanding of the intricate interactions between parasites, hosts, and the microbiome is essential to develop effective strategies for preventing and treating chronic inflammatory diseases. SCFA, a short-chain fatty acid metabolite. This figure was created using BioRender.com.

Autoimmune diseases have a multifactorial origin, with genetics playing an important role in determining predisposition (Patsopoulos et al., 2019; Harroud and Hafler, 2023); however, what some have termed as an ‘autoimmune epidemic’ can’t be solely attributed to gradual genetic changes, which means that environmental factors play a decisive role. Several environmental agents have been implicated, including dietary factors, plant, animal, and house environment, vitamin D levels, viral infections, and the gut biome, among others, (Zhao et al., 2019; Sundaresan et al., 2023; Sîrbe et al., 2022; Zhang et al., 2020); see Figure 1.

The microbiota maintains gut and systemic immune homeostasis by modulating its innate and adaptive branches. It achieves this through two main pathways: the production of metabolites and the release of bacterial-derived factors that directly interact with the host’s immune system (Zheng et al., 2020). There are three major classes of microbiota-derived metabolites: short-chain fatty acids (SCFAs), tryptophan (Trp), and bile acid (BA) metabolites (Wang et al., 2023b). SCFAs, carboxylic acids with less than six carbon atoms, are produced by bacteria as a result of carbohydrate fermentation. The most common SCFAs are acetate, propionate, and butyrate. Through regulation of epigenetics and binding to membrane receptors, they have been shown to modulate the immune system and inflammation by reducing the recruitment of innate immune cells and the differentiation of lymphocytes (Yao et al., 2022), see Figure 1. Trp derives from ingested proteins and is transformed into different metabolites by the gut microbiota, including indole, indole-3-acid-acetic, indole-3-propionic acid, indoleacrylic acid, and tryptamine. Several of these metabolites contribute to gut homeostasis and exert anti-inflammatory functions through binding to nuclear receptors: the aryl hydrocarbon receptor (AhR) and the pregnane X receptor (PXP) (Su et al., 2022). BA is synthesized in the liver and transported to the intestine, where they are transformed by bacteria, generating BA metabolites that regulate metabolic and immunological function (Cai et al., 2022). An imbalance in the levels of microbiota-derived metabolites due to dysbiosis has been associated with a wide range of metabolic and inflammatory diseases (Hand et al., 2016; Parada Venegas et al., 2019; Ma et al., 2024). As a result, in recent years, there has been a considerable focus on the development of microbiome-targeted interventions to restore eubiosis through various approaches, including prebiotics, probiotics, dietary modifications, and fecal matter transplants.

In addition to dietary patterns, physical activity, and antibiotics, helminth infection is another factor that could induce a shift in microbiome composition (Figure 1). Findings on this topic have been somewhat inconsistent, with some studies reporting changes in microbial diversity and others indicating no significant effects. A study conducted in a rural village in the Philippines found that helminth-positive individuals increased *Faecalibacterium* abundance and that the increase was more pronounced in subjects co-infected with several helminth species (Gordon et al., 2020). Notably, it has been reported that *Faecalibacterium* species have anti-inflammatory properties, promoting the induction of regulatory T cells (Treg), and are decreased in patients with inflammatory bowel diseases (Touch et al., 2022). Another study compared the microbiome of indigenous Malaysians from five villages with different prevalences of helminth infections and found that those living in villages with a higher rate of helminth infections had more diverse microbiomes and more uncharacterized bacterial species, and in the different villages, the helminth infection was associated with the differential abundance of distinct bacterial taxa; interestingly, after deworming with albendazole there were no significant changes in species richness (Tee et al., 2022).

The variability in the results obtained so far could be due to differences in helminth species and their anatomical site of infection, stage of infection, geographic location, host genetics, lifestyle, and methodological approaches. Given the implication of the gut biome in the development of chronic inflammatory diseases and the ability of helminths to induce a state of immune hyporesponsiveness and regulatory networks in the host to benefit themselves, it’s crucial to deepen our understanding of the complex interactions of parasites, host and the microbiome (Figure 1).

## Helminths and immune regulation

Helminths are organisms with complex life cycles, living up to decades within their hosts. Their life cycle usually involves migratory phases during the different stages of infection, resulting in damage to several host tissues. The prototypical immune response to a helminth infection is characterized by type 2 immunity, involving T helper 2 cells (Th2), IgE production, eosinophils, mast cells, basophils, alternatively activated macrophages, type 2 innate lymphoid cells (ILC2) and the cytokines IL-4, IL-5, IL-9 and IL-13, among others. This response promotes the expulsion of the parasite and tissue repair pathways (Maizels and Gause, 2023; Smallwood et al., 2017). To be able to establish chronic infections and promote their survival within the host, helminths have developed an array of immunoregulatory pathways, characterized by an expansion of Treg, elevated levels of IL-10 and TGFβ, immunoregulatory monocytes and B-cell class switch to IgG4, associated with immune tolerance (Maizels et al., 2018; Gazzinelli-Guimaraes and Nutman, 2018). A balance between the Th2 and the Treg responses is likely to be essential to allow the survival of both host and parasite since an excess of either would damage the host due to pathological fibrosis or severe parasite burden (Harris and Loke, 2017; Everts et al., 2010). Considering also that helminth infections are associated with changes in the microbiome and that its composition plays a significant role in the development and progression of inflammatory diseases, as discussed earlier, it is plausible that helminths also induce immunomodulation in the host by altering microbial diversity (Llinas-Caballero et al., 2022; Kupritz et al., 2021). For example, it was recently demonstrated that infection with an extra-intestinal helminth, *Echinococcus multilocularis,* attenuates colitis in mice through the expansion of colonic Tregs. This effect was partially mediated by a shift in microbiome composition, resulting in elevated levels of SCFAs (Wang et al., 2023a).

Helminths have evolved sophisticated mechanisms to modulate the host’s immune system, with ESP being the primary one. These ESP comprise all the molecules that are actively released or produced as waste throughout their life cycle, including proteins, small peptides, enzymes, lipids, and glycans, and they are specific in species and stage (Maizels et al., 2018; van der Zande et al., 2019; Sobotková et al., 2019). In the last decades, scientists have invested significant effort in identifying and isolating bioactive molecules released by helminths due to their potential to be developed as therapeutic agents. A promising candidate for an ESP-based therapeutic is the TGF-β mimic from the murine helminth parasite *Heligmosomoides polygyrus* (Hp-TGM). This molecule, which mimics endogenous proteins, exhibits potent immunoregulatory properties, including the expansion of Tregs (Johnston et al., 2017). Interestingly, intranasal and parenteral administration of Hp-TGM has successfully alleviated allergic airway inflammation in mice, demonstrating its capacity to control lung inflammation and allergic pathology (Chauché et al., 2022). More recently, a paradigm shift occurred after discovering that ESP contains EVs, allowing the helminth to deliver fragile cargo, like RNA, to distant cells (Marcilla et al., 2012).

The concept of ‘biome repletion’ arose as a potential strategy to address the rising prevalence of inflammatory, allergic, and autoimmune diseases and advocate for the integration of live microbiota into standard clinical approaches, including the exploration of helminth administration in clinical trials (Rook, 2023; Bilbo et al., 2011) or the development of helminth-derived products that target specific immune response (Maizels et al., 2018; Ditgen et al., 2014). Several studies in mice with experimental autoimmune encephalomyelitis (EAE), an animal model of multiple sclerosis (MS), have explored the effects of helminth therapy. Preimmunization with eggs of the trematode *Schistosoma mansoni* decreased immune cell infiltration into the central nervous system and improved clinical scores, with these effects being dependent on STAT6, a key mediator in Th2 differentiation (Sewell et al., 2003). EAE disease severity was also ameliorated by *H. polygyrus*, in an IL-4Rα-dependent manner, and that protection requires live helminth infection. It is essential to note that Hp-TGM does not fully replicate the suppressive effects of live *H. polygyrus* infection indicating that additional regulatory pathways are activated by infection, which could be crucial for EAE (White et al., 2020). In this model, disease protection is associated with increased Tregs, GATA3 +, and ST2 + cells, reduced RORγt+and IL-17A cell responses, and a lower level of myeloid cell infiltration into the CNS (White et al., 2020).

In contrast, another study analyzed the effects of infection with the nematode *Strongyloides venezuelensis* on the development of EAE in mice and found no alterations in clinical score or inflammation in the central nervous system (Chiuso-Minicucci et al., 2011), whereas infection with the nematode *Toxocara canis* worsened the course of the disease, resulting in higher clinical scores and increased weight loss (Novák et al., 2022). These studies demonstrate the complex interactions between different helminths and the host in the context of inflammatory diseases and highlight the importance of certain helminth species for potential therapeutic applications, as well as the dosage, timing of inoculation, and life stage of the parasite. Despite the variation of protocols, most of the research has shown a protective effect of helminths in the context of EAE, especially when administered before the onset of disease (Charabati et al., 2020). Further research is needed to elucidate the specific regulatory pathways underlying each disease and to identify appropriate ESP candidates for the development of more specific treatment strategies.

Previously, pioneer work in the field studied a cohort of patients with MS in Argentina with naturally-acquired gastrointestinal helminth infections were followed for 5 y and showed a significantly lower number of relapses, reduced disability scores, and lower Magnetic Resonance Imaging (MRI) activity compared to uninfected MS patients. Notably, when four of these patients received anthelmintic treatment, their disability scores and their exacerbations significantly increased, providing a direct role of the helminths in the modulation of this autoimmune disease (Correale and Farez, 2007; Correale and Farez, 2011). Furthermore, our group demonstrated that these natural helminth infections enhanced a negative regulatory axis, as the tyrosine kinase TYRO3, AXL, and MERTK (TAM) receptors and their ligands (PROS1 and GAS6), contributing to the dampening of the inflammatory response in patients with MS (Ortiz Wilczyñski et al., 2020). In this study, we found first a reduced levels of TYRO3 in circulating monocytes, dendritic cells, and CD4 lymphocytes in patients with only MS, compared to healthy donors, denoting a strong influence of the disease condition. Remarkably, this scenario is reversed in patients with MS who have naturally acquired gastrointestinal helminth infections, increasing the expression of three TAM receptors in antigen-presenting cells and their agonist GAS6 in circulating monocytes and CD4 + T cells compared to uninfected MS counterparts. PROS1 expression was also enhanced on circulating monocytes under the Th2-helminth environment. Interestingly, CD4 + IL-10+-producing cells from MS patients with helminthic infection showed higher levels of GAS6 expression than Th17 cells, and GAS6 blockade induced an expansion of Th17 effector genes. Furthermore, adding recombinant GAS6 into activated CD4 + T cells from MS patients restrains the Th17 gene expression signature. This cohort of patients with naturally occurring helminthic infection unravels a promising regulatory mechanism to control the Th17 inflammatory response in autoimmunity (Ortiz Wilczyñski et al., 2020; Carrera Silva et al., 2025). Altogether, we can speculate here that the helminth-type 2 environment along with parasite-derived products, may be training innate and adaptive immune cells as well as bone marrow precursors.

Almost 20 y of human clinical trials have explored the use of experimental helminth infections to treat inflammatory and autoimmune diseases, including celiac disease, inflammatory bowel disease (IBD), MS, rheumatoid arthritis (RA), and psoriasis, based on their masterful skill to modulate immune responses. Thus far, some of these trials have established that therapy is safe with some evidence of therapeutic effect (Ryan et al., 2020). However, discordance in mouse-to-human translation is a well-known challenge in various research fields. Of note, clinical trials in this area have been hindered by limitations such as small sample sizes, lack of some control groups, and the use of few species as well non-human-tropic helminths. We will discuss in more detail some specific cases of helminth impact on human inflammatory diseases in a specific section below.

## Trained immunity by helminths

The remarkable ability of parasites to subvert host immunity stems from their manipulation of key immune components: CD4 + T cell differentiation, Treg cell induction, B switching, and Breg cells (Smallwood et al., 2017; Maizels et al., 2018). This intricate interplay between host and parasite drives a complex immune response. However, despite its potential significance, the nature and mechanisms of helminth-trained immunity remain relatively unexplored. This phenomenon may be fundamental to understanding regulatory mechanisms and tissue repair pathways that do not compromise acquired immunity while preventing excessive inflammation and autoimmune diseases. Trained immunity can influence T cell fate and function, which in turn can modulate innate cell activity. The initial insult determines the balance between pro- and anti-inflammatory responses within this bidirectional synapse, the cytokine microenvironment, and the specific cellular functions induced by trained immunity (Murphy et al., 2021).

**Figure 2.**
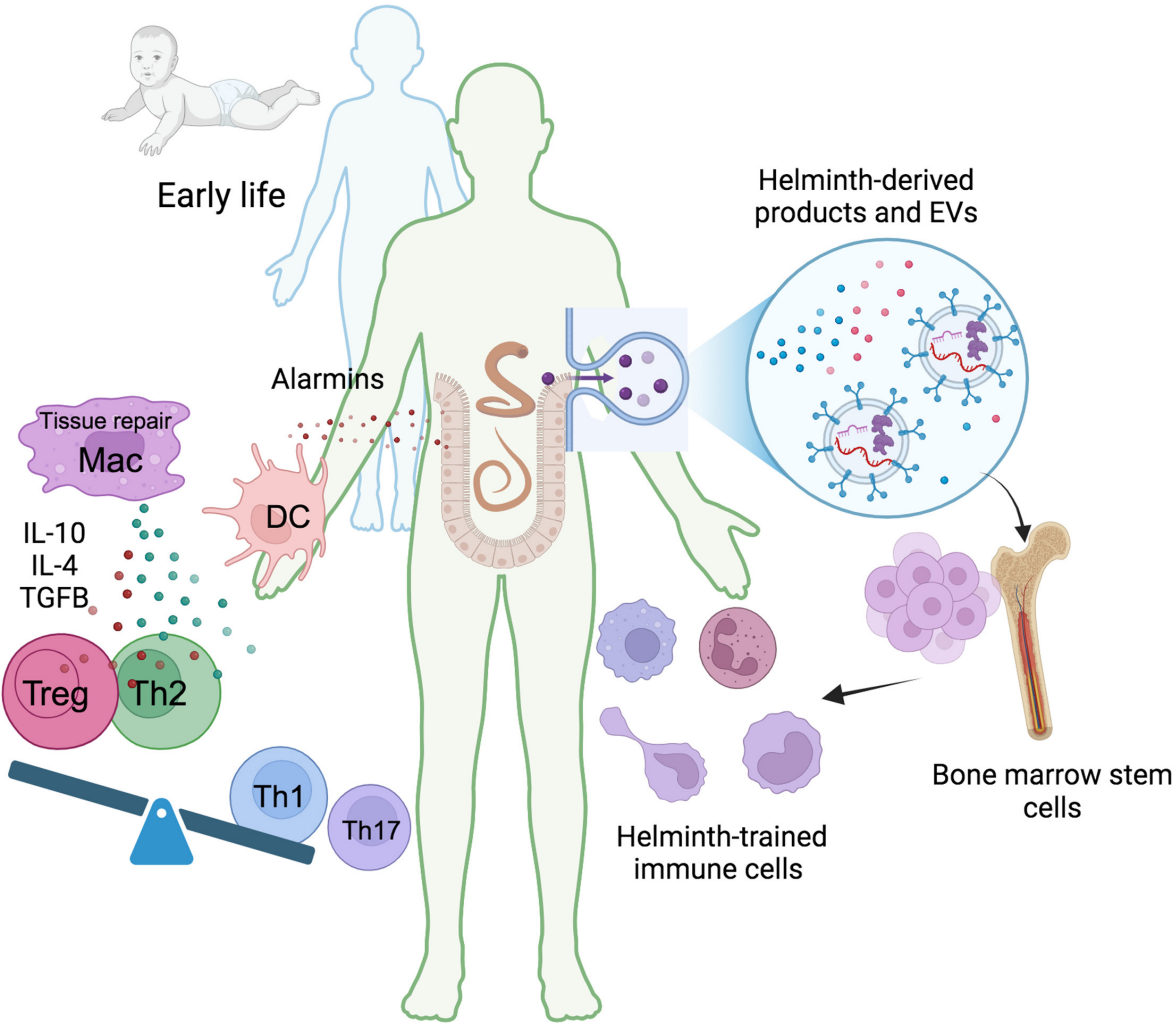
Helminth-induced trained immunity. Early life immune education is crucial for developing a robust and well-regulated immune system. Humans have evolved a symbiotic relationship with commensal microbiota, and disruptions to this balance can lead to immune dysregulation. Helminths, with their complex life cycles and prolonged host interactions, can significantly influence host immunity. During infection, tissue damage caused by migrating helminths triggers the release of alarmins like TSLP, IL-25, and IL-33. These alarmins recruit monocytes and DC, and the Th2 environment promotes the differentiation of anti-inflammatory and tissue repair macrophages. These trained macrophages produce higher levels of IL-10, IL-4, or TGFβ promoting the differentiation of regulatory T cells (Tregs) and suppressing pro-inflammatory Th1 and Th17 responses. Helminth-derived products, including small peptides, enzymes, lipids, and EVs carrying various molecules like RNA and proteins, can induce central anti-inflammatory trained immunity. This leads to the generation of long-lasting anti-inflammatory myeloid cells, suggesting a potential impact on bone marrow hematopoietic progenitors. TSLP, thymic stromal lymphopoietin; DC, dendritic cells; Mac, macrophages; Treg, regulatory T cells; EVs, extracellular vesicles. This figure was created using BioRender.com.

While most studies have described trained immunity as enhancing pro-inflammatory responses, recent findings indicate that myeloid cells can be trained towards an anti-inflammatory phenotype following exposure to helminth products. Macrophages trained in vitro with *Fasciola hepatica* total extract (FHTE) exhibited increased production of the anti-inflammatory cytokines IL-10 and IL-1RA while concomitantly decreasing the production of the pro-inflammatory cytokines TNF and IL-12p40 (Murphy et al., 2021; Quinn et al., 2019). The increased IL-10 could be promoting Tregs differentiation, while IL-1RA suppresses the Th17 response. The reduced production of IL-12p40 and TNF may also dampen the Th1 cell response Figure 2.

Zakeri et al., 2022 demonstrated that soluble products from the nematode *Trichuris suis* (TSP) induce a trained phenotype on bone marrow-derived macrophages (BMDM) that restrains inflammatory responses. Treatment of BMDMs with TSP for 24 hr and subsequent stimulation with TLR agonists after 72 hr increased IL-10 secretion compared with untreated BMDMs. In contrast, IL-6 secretion was decreased in response to all TLR agonists except one, while TNF secretion yielded mixed results depending on the agonist used. Metabolic flux analyses revealed that TSP-treated BMDMs had a higher mitochondrial oxygen consumption rate, indicating increased oxidative phosphorylation than untreated BMDMs. These results indicate that TSP has the ability to reprogram macrophage metabolism and induce anti-inflammatory trained immunity (Zakeri et al., 2022).

Interestingly, Quinteros et al*.* demonstrated that a peptide derived from the trematode *Fasciola hepatica*, FhHDM-1, could reverse the inflammatory trained phenotype of BMDMs from non-obese diabetic (NOD) mice (Quinteros et al., 2024). BMDMs from NOD mice showed epigenetic signatures of trained immunity, as well as high glycolytic activity and enhanced secretion of IL-6 and TNF in response to LPS when compared to BMDMs from control mice, suggesting that progenitor cells in the bone marrow of NOD mice had immune training. Administration of intraperitoneal injections of FhHDM-1 and posterior analysis of BMDMs revealed a reduction in glycolytic activity in response to LPS in contrast to NOD mice not treated with the helminth product. The secretion of IL-6 and TNF was reduced to the levels observed in control mice, as well as the epigenetic modifications previously mentioned. This emerging understanding of regulating inflammatory responses through trained immunity induced by helminths or their products has significant potential for therapeutic strategies in treating inflammatory and autoimmune disorders Figure 2.

In this regard, Mills and colleagues have made significant discoveries in this field (Quinn et al., 2019; Cunningham et al., 2021). Their research demonstrated that treating mice with *F. hepatica* ESP induces an anti-inflammatory phenotype in bone marrow hematopoietic stem cells (HSC). This, in turn, leads to an expansion of the peripheral population of Ly6C^low^ monocytes, associated with anti-inflammatory activity, and the generation of BMDMs that secrete more anti-inflammatory cytokines and less pro-inflammatory cytokines after stimulation with various TLR agonists. This confirms the capacity of helminths ESP to induce central anti-inflammatory trained immunity that gives rise to anti-inflammatory circulating myeloid cells. More importantly, this mechanism provides protection against EAE through the generation of anti-inflammatory monocytes that suppress T-cell mediated autoimmunity, and this effect persists for at least 8 mo (Cunningham et al., 2021), see also Figure 2.

Trained immunity in Th2 responses has also been studied, and Yasuda et al*.* found that infection by the nematode *Strongyloides venezuelensis* induced resistance to a subsequent infection by *Nippostrongylus brasiliensis*, an unrelated nematode, through innate cell-dependent mechanisms in mice (Yasuda et al., 2018). This effect lasted at least 3 mo and was mediated by training ILC2s in the lung, a tissue through which the larvae migrate. In vitro stimulation of these cells with PMA and ionomycin, two potent cell activators, led to enhanced production of the cytokines IL-5 and IL-13 in ILC2s derived from mice that had been infected with *S. venezuelensis*. After the infection with *N. brasiliensis*, the expression of Il13 and Irf4 mRNAs was significantly higher in ILC2s from mice that had been previously infected with *S. venezuelensis* compared to uninfected mice. These findings suggest that ILC2s have the capacity to acquire a trained phenotype that persists over time in response to helminth infection and contributes to the host’s resistance in an antigen-independent manner.

Furthermore, helminths induce a systemic innate mucin response that primes peripheral barrier sites for protection against subsequent secondary helminth infections. Mechanistically, a cross-mucosal immune mechanism by which intestinal helminths (*Trichinella spiralis* or *Heligmosomoides polygyrus*) may protect their hosts against co-infection by a different parasite (*N. brasiliensis*) at a distal site via ILC2s in the gut and circulation of activated CD4 + T cells that can be triggered to release effector cytokines and mount inflammatory responses by tissue damage-associated alarmins, such as IL-33 priming (Campbell et al., 2019; Filbey et al., 2019; Löser et al., 2019).

Helminth-derived products, such as EVs, may play a pivotal role in training immune cells. However, the cellular source of EVs, and their route of secretion from the helminth is still not fully characterized. While understanding the source and biosynthesis of helminth EVs is crucial, a more pressing question is their physiological function. Are these EVs released incidentally as a byproduct of parasite processes, or do they serve a specific purpose in manipulating the host immune response? (Drurey and Maizels, 2021; Sotillo et al., 2020; Coakley et al., 2016). EVs influence a wide range of biological systems, including mammalian immunity, and emerging evidence suggests they play a role in immune regulation (Kalluri and LeBleu, 2020; Zhou et al., 2020). EVs serve as a crucial mode of communication between innate and adaptive immune cells. Given their role in immune signaling, it is not surprising that pathogens have evolved to exploit EVs for immunosuppression. The malaria parasite, *Plasmodium berghei*, secretes EVs that can inhibit T-cell responses (Demarta-Gatsi et al., 2019). *Leishmania donovani* exosomes have been found to push monocytes and dendritic cells towards anti-inflammatory phenotypes, increasing Th2 polarisation and thereby contributing to the disease (Silverman et al., 2010).

The composition of helminth-derived EVs varies depending on the gender and life cycle stage of the parasite. While the primary cargo typically includes lipids, nucleic acids, and proteins, the potential role of these EVs in host immune regulation suggests the presence of some components beyond the standard EV repertoire. Most analyses of helminth EV content have focused on the proteome (Sotillo et al., 2020). However, helminth EVs also contain various small RNA species, particularly microRNAs (miRNAs), which have been implicated in host-helminth interactions and provide some of the most significant clues for immunomodulation (Sotillo et al., 2020; Wu et al., 2018; Fromm et al., 2017; Hansen et al., 2019; Yang et al., 2020). Lipids represent another significant component of EVs, although they are less well-characterized than proteins and small RNAs. Lipids can possess bioactivity, directly influencing cell uptake and modulating immune responses (Assunção et al., 2017; Magalhães et al., 2018). Glycans are another neglected group within helminth EV biology. Both EV proteins and lipids can be post-translationally glycosylated, which is known to be essential for the regulation of protein function. Glycans have been identified as key players in the regulation of EV uptake, affecting the cell tropism of EVs. Glycans may even play a role in the biodistribution of helminth EVs (Drurey and Maizels, 2021; Whitehead et al., 2020; de la Torre-Escudero et al., 2019).

Most helminth-derived EVs are collected from parasites cultured in vitro and purified from conditioned media. Only some exception involves harvesting EVs from the hydatid cyst fluid of *E. granulosus* and comparing them with those generated by the protoscolex stage of the parasite in vitro (Zhou et al., 2019; Chop et al., 2024; Rodriguez Rodrigues et al., 2021).

Phagocytes, particularly macrophages, have been central to helminth EV research due to their plasticity in transitioning from a pro-inflammatory to an anti-inflammatory and tissue repair phenotype. Similarly, circulating myeloid cells, such as monocytes and adaptive lymphocytes, can be reprogrammed by EVs at distant sites (Drurey and Maizels, 2021). Intriguingly, recent studies suggest that trained immunity may even originate at the hematopoietic stem cell stage, (Quinn et al., 2019; Cunningham et al., 2021), see also Figure 2.

Helminth-derived EVs can also exert a localized immune impact, such as influencing intestinal epithelial cells as Tuft cells by regulating the release of alarmins like thymic stromal lymphopoietin (TSLP), IL-25, and IL-33. Additionally, helminths can promote tissue infiltration by releasing cathepsins and other proteases. Similarly, the migration of hookworm larvae through the lung causes significant mechanical and enzymatic damage, but rapid tissue repair is observed, potentially facilitated by EVs to restore homeostasis (Drurey and Maizels, 2021).

**Figure 3.**
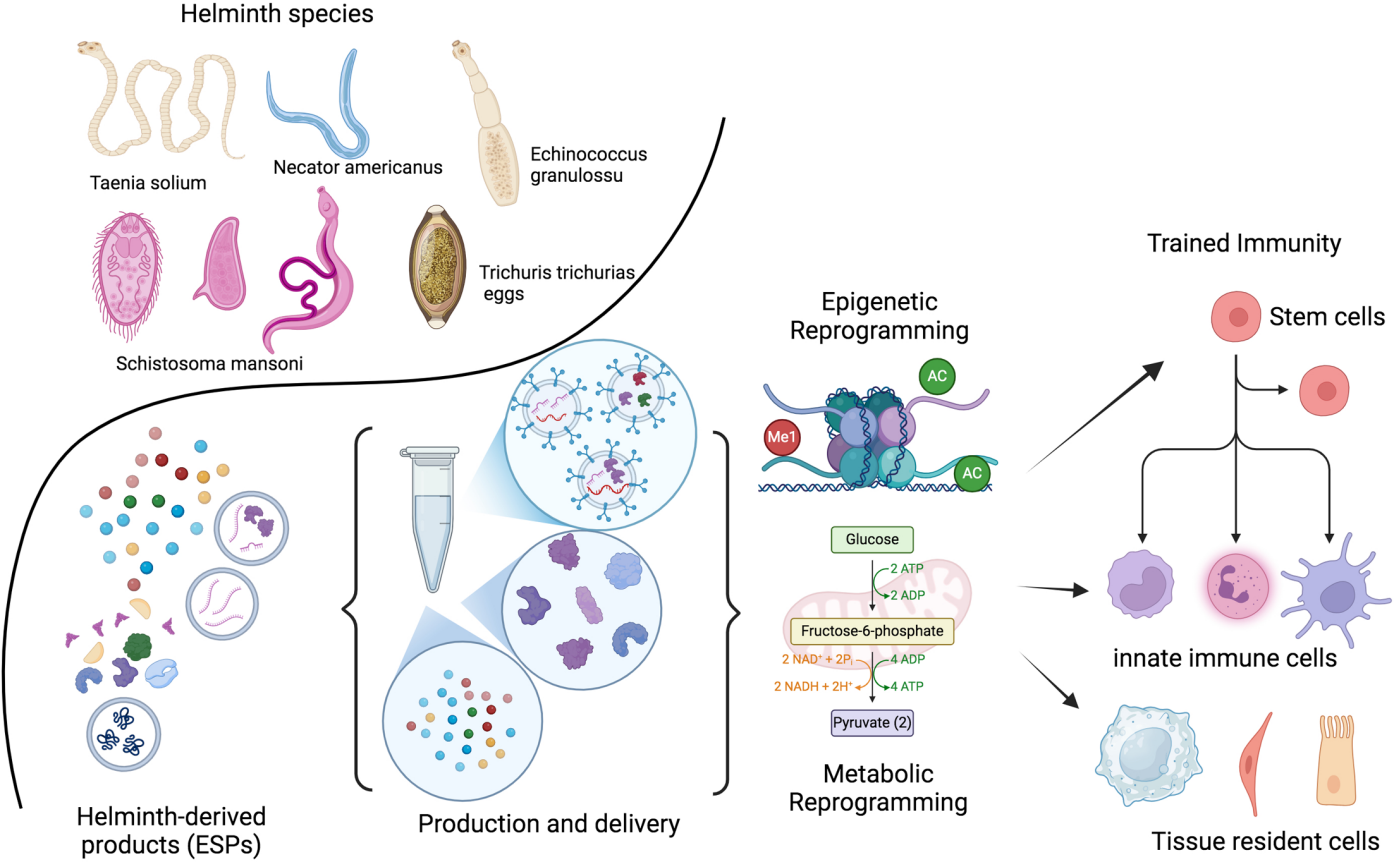
Therapeutic potential of helminths and their products. The therapeutic potential of helminths and their products depends on various factors, including the specific helminth species, the dosage, timing of inoculation, and the parasite’s life stage. Helminths have evolved sophisticated mechanisms to modulate the host immune system. Their products (ESPs) contain a diverse range of molecules, including proteins, peptides, enzymes, lipids, glycans, and EVs. These EVs can deliver fragile cargo, like RNA, to distant cells. Identifying and characterizing these molecules and their target pathways presents a unique opportunity to develop novel, safe, and effective therapeutic strategies inspired by nature. Recent research suggests that the adaptation of the developing immune system to helminths involves epigenetic and metabolic changes. These adaptations may be lost after a few generations without helminth exposure. The goal is to identify the optimal combination of patient, genetic factors, disease, and helminth products or their metabolic byproducts to train the immune system both locally and at the stem cell level. ESPs, excretory/secretory products; EVs, extracellular vesicles. This figure was created using BioRender.com.

Tuft cells have been identified in intestinal and lung tissues (Haber et al., 2017) as rare chemosensory epithelial cells that monitor their environment and relay messages to the surrounding tissue via secretion of neuro- and immunomodulatory molecules (Strine and Wilen, 2022). These cells have evolved to perform critical functions in modulating host-microbe interactions, but a critical question is whether tuft cells can be trained (Chen et al., 2024). Based on their known signaling pathways, tuft cells have been linked to a wide variety of bodily functions, such as the establishment of T cell tolerance (Miller et al., 2018), cross-talk with the nervous system (Matsumoto et al., 2011), and epithelial repair and remodeling (Huang et al., 2024). Given these roles, it is unsurprising that tuft cells have been implicated in IBD as well as contributing to helminth clearance by activation of the tuft-ILC2 circuit that results in rapid remodeling of the intestinal epithelium, and protective functions of IBD manifestations (Yi et al., 2019; Kjærgaard et al., 2021; Qu et al., 2015; Banerjee et al., 2020). The specific mechanisms by which tuft cells mitigate IBD have not been elucidated, but this may be microbiome dependent, increased expression of genes that regulate the tricarboxylic acid cycle, which resulted from microbe production of the metabolite succinate Figure 3. Finally, while intestinal tuft cells are recognized for their crucial roles in the host defense against intestinal pathogens, there remains uncertainty regarding their trainability. In this sense, an interesting work by Chen et al., 2024 showed that trained immunity of intestinal tuft cells during infancy enhances host defense against enteroviral infections in mice. The authors showed that tuft cells can be trained by IL-25, which supports faster and higher levels of IL-25 production in response to enteroviral infection and further exhibits an anti-enteroviral effect (Chen et al., 2024).

Research on helminth-induced trained immunity in humans is limited. However, evidence suggests certain stimuli can induce centrally trained immunity in humans. For example, BCG vaccination has been shown to reprogram hematopoietic stem cells (HSCs) at the transcriptomic, epigenetic, and functional levels, generating trained monocytes for at least 3 mo (Cirovic et al., 2020; Sun et al., 2024). Despite this progress, our understanding of how specific drivers of trained immunity differentially affect human T cell subsets and disrupt the delicate balance between Treg/Th17 or Th1/Th2 cells remains limited.

We certainly need to move forward in how helminth-derived products as EVs interact with the human immune system, particularly considering the long-lasting and often asymptomatic nature of helminth infections, which are associated with widespread immune downregulation.

## Impact on human inflammatory diseases

Chronic inflammatory and autoimmune disorders are characterized by the immune system’s assault on its own tissues, affecting millions worldwide. With over 80 recognized autoimmune diseases, including IBD, MS, RA, and type 1 diabetes (T1D), the global health (10%) and economic burden is immense. Addressing this growing epidemic requires innovative approaches to prevent and treat autoimmune diseases (Smallwood et al., 2017; Gutierrez-Arcelus et al., 2016; Hayter and Cook, 2012). Although genetic predisposition influences susceptibility, some environmental and lifestyle factors are likely crucial in triggering or exacerbating these conditions as dysbiosis and loss of helminths interaction, which promote immunoregulatory mechanisms that could benefit the host by protecting them from severe inflammatory pathologies (Rook, 2023; Scudellari, 2017; Smallwood et al., 2017). Many micro and macro organisms responsible for regulating the immune system are derived from our mothers, families, and the natural environment (including animals), see Figure 1. These organisms, often symbiotic members of a healthy host biome, contribute to a balanced immune response (Rook, 2023; Scudellari, 2017).

Type 2 immune responses, rooted in ancient defense mechanisms, play a crucial role in protecting against metazoan endo- and ectoparasites, regulating metabolism, and promoting tissue repair. While these responses are essential for health, dysregulation can lead to allergic disorders, impaired tissue healing, and metabolic disturbances (Maizels and Gause, 2023; Gause et al., 2013). The diversity of these macroparasites and their manifold evasion strategies has demanded a corresponding diversification of defense mechanisms for host survival that are fine-tuned to each threat (Maizels and Gause, 2023). Most attention in recent years has been given to the pathways of type 2 induction and its regulation in immunological disorders (Gause et al., 2020; Hammad and Lambrecht, 2015; McDaniel et al., 2023).

It was once logical to postulate evolved dependence on helminths in the past (Bilbo et al., 2011; Maizels, 2020); however, it now seems more likely that the adaptation of the developing immune system to the presence of helminths was largely epigenetic and was lost after a few generations without them. These reversible epigenetic mechanisms help us to understand the conflicting and disappointing results of helminth therapy trials. Clinical trials may not yield helpful results, and face significant challenges until we can precisely identify the optimal combination of patient characteristics, genetic factors, disease stage, and specific helminth species, ESPs, or metabolic byproducts for effectively train immune system (Rook, 2023; Smallwood et al., 2017; Ryan et al., 2020; Loke et al., 2022). To mitigate risks associated with live parasite infections, exploring helminth-derived anti-inflammatory molecules or ESPs delivered through EVs offers a promising avenue for developing safer and more controlled therapeutics for chronic inflammatory diseases.

Completed and ongoing therapeutic clinical trials using helminth products in various human disease contexts are summarized by Ryan et al in their Table 1 (Ryan et al., 2020), focusing on safety, tolerability, and efficacy. Some examples of the therapeutic potential of helminth exposure or helminth-derived products for conditions like IBD, MS, and asthma are discussed below.

*Inflammatory bowel diseases (IBD)*, comprising Crohn’s disease (CD) and ulcerative colitis (UC), are chronic, progressive, inflammatory conditions of the gastrointestinal tract. Nowadays, it is clear that an imbalance in the gut microbial community, or dysbiosis, represents a critical environment factors, that results in IBD. Several animal models have shown the beneficial effect of helminth infections or their products on the microbiota and the immune regulation of IBD (Atagozli et al., 2023; Shi et al., 2022; Ryan et al., 2022; Rapin et al., 2020). Thus, IBD-associated dysbiosis, marked by a loss of beneficial Bacteroides and Firmicutes and an increase in pro-inflammatory Enterobacteriaceae, is a key feature of the disease. Within this IBD-microbiota-dysbiosis framework, helminths are of increasing interest due to their capacity to modulate gut microbiota composition, enhance cecal bacterial diversity, and ameliorate IBD in animal models. A novel discovery and validation pipeline, detailed by Ryan et al., 2022, led to the identification of numerous anti-inflammatory biologics from the recombinant secretome of gut-dwelling hookworms. These proteins, representing distinct families, demonstrated protective effects against inducible colitis in mice, suggesting they are safe and promising drug candidates.

Deworming trials have revealed an increased likelihood of developing various autoimmune and metabolic diseases with the use of antihelminthic drugs. This underscores the importance of considering the evolutionary context of human-parasite interactions and the potential risks of disrupting these ancient relationships (Tahapary et al., 2017; Sanya et al., 2020; Flohr et al., 2010; Shute et al., 2021). In this sense, Shute et al., 2021 demonstrates the critical role of bacterial SCFAs via free fatty-acid receptor-2 (ffar2) in *H. diminuta*-induced colitis improvement, the necessity of IL-10 in upregulating SCFA transporters/receptors, and butyrate’s regulation of IL-10 receptor expression. The findings suggest that the failure of helminth therapy in some IBD trials may be due to patient-specific deficiencies in SCFA production, transport, or IL-10 signaling.

Several clinical trials have been run for over 15 y and have yielded early promising results but also some disappointing outcomes (Ryan et al., 2020; Atagozli et al., 2023). Furthermore, recent systematic reviews summarized the results of these studies into two categories: (a) the efficacy of helminth therapy and (b) the safety of helminth therapy. Results regarding the efficacy were mixed, and a conclusive answer could not be reached, as there was not enough evidence to rule out a placebo effect. Despite this, helminth therapy was safe and tolerable (Ryan et al., 2020; Alghanmi et al., 2024; Shields and Cooper, 2022; Axelrad et al., 2021). Nonetheless, epidemiological explorations, basic science studies, and clinical research on helminths can lead to the development of safe, potent, and novel therapeutic approaches to prevent or treat IBD (Ryan et al., 2020; Ryan et al., 2022; Shute et al., 2021; Maruszewska-Cheruiyot et al., 2023).

*Multiple sclerosis (MS)* is a highly disabling neurodegenerative autoimmune condition in which an unbalanced immune response plays a critical role. The etiology of MS remains elusive, but it is now known that environmental and patient-specific factors and susceptible genes (more than 200 autosomal vulnerability variants) were associated with disease pathogenesis. Accumulating evidence suggests that the clinical course of multiple sclerosis is better considered as a continuum, where both inflammatory and neurodegenerative processes occur in all disease courses and cannot be clearly assigned to separate, sequential disease stages (Kuhlmann et al., 2023; Correale et al., 2017). Accurate diagnosis of MS can be complex in populations from Latin America, Africa, the Middle East, eastern Europe, southeast Asia, and the Western Pacific. Unique environmental exposures, genetic predispositions, and varying healthcare access in these regions can significantly influence disease presentation and diagnostic criteria (Correale et al., 2024). One of the most striking illustrations of the importance of the environment in MS pathogenesis is its geographic distribution; prevalence rates are increased in high-latitude regions yet uncommon near the equator (Melcon et al., 2014). The gut biome is recognized as a critical regulator of immune and nervous system function, significantly impacting both the onset and progression of MS. It may contribute to and influence the production of soluble metabolites and immune and neuroendocrine factors (Correale et al., 2022). The potential role of epigenetics may explain the inconsistent outcomes of helminth therapy trials in MS (Charabati et al., 2020; Ryan et al., 2020). While natural helminth infections in childhood, more frequent in South American regions like Argentina (Correale and Farez, 2007; Correale and Farez, 2011), can halt disease progression, therapeutic helminth administration in populations without a history of such infections has yielded disappointing results (Ryan et al., 2020; Tanasescu et al., 2020). The treatment of relapsing MS patients with larvae of the nematode *Necator americanus* was proved to be safe and well tolerated and induced a significant increase in peripheral blood Tregs. Still, the number of new/enlarged brain lesions was not different from the placebo group (Tanasescu et al., 2020). Similarly, results were obtained using eggs from the nematode *Trichuris suis*, where MS patients tolerated the helminth infection, but it didn’t show beneficial effects (Voldsgaard et al., 2015). In other recent study, MS patients receiving TSO treatment established a *T. suis*-specific T- and B-cell response; however, with varying degrees of T cell responses and cellular functionality across individuals, which might account for the overall miscellaneous clinical efficacy in the studied patients (Yordanova et al., 2021).

*Asthma and allergic airway inflammation* are described as IgE-mediated diseases, characterized by a Th2-driven inflammation where environmental exposures and host factors synergistically contribute to its pathogenesis. The escalating global prevalence of these conditions (20%) is a consequence of complex gene-environment interactions that modulate the immune system, and are strongly linked to modern westernized lifestyles (Murrison et al., 2019). A substantial body of evidence obtained from experimental studies in mice points towards a protective role due to the regulatory pathways induced by the parasites that help counteract the immune hyperresponsiveness present in allergy and asthma (Smits et al., 2010). Moreover, several ESP derived from different helminth species have been shown to reduce or suppress allergic airway eosinophilia and inflammation in mice (Chauché et al., 2022; Zhang et al., 2019; Pitrez et al., 2015; Xu et al., 2025). However, other studies have shown an exacerbation of allergic asthma in response to the administration of helminth antigens, unveiling the complexity of the interaction between these type 2 immunity inducers (Ghabdian et al., 2022). On the other hand, the evidence on humans remains more conflicting. As seen with autoimmune diseases, epidemiological data suggests an inverse correlation between helminth infections and those of asthma and allergy (Logan et al., 2018); however, when considering the parasite species, some like *A. lumbricoides* have been associated with an increased risk of asthma and disease severity (Cruz et al., 2017; Arrais et al., 2022). Clinical trials have been conducted to assess the efficacy of helminth therapy for asthma and allergic rhinitis, using *N. americanus* and *T. suis* ova, respectively. Even though they were proven to be safe and well tolerated, clinical benefits were minimal. It is speculated that the discrepancy between human and animal studies could be explained by the fact that helminths are effective at preventing the development of allergy, not treating it (Evans and Mitre, 2015). Despite the lack of positive results in humans thus far, further investigations with improved approaches and protocols are necessary for the treatment of type 2 inflammatory diseases.

Several inflammatory diseases are associated with the biome depletion theory, and increased inflammatory diseases and helminth-mediated protection are not restricted to autoimmune and allergic diseases (Smallwood et al., 2017; Maizels, 2020). There is an inverse relationship observed between human helminth infection, insulin resistance, and type 2 diabetes, and it has been proposed that chronic helminth infection results in long-term beneficial effects on host metabolism, especially on white adipose tissue, intestines, and liver (van der Zande et al., 2019; Wiria et al., 2014).

In developed countries, the successful application of helminth therapy may ultimately depend on precise patient selection and the careful matching of specific helminth species (Rook, 2023; Smallwood et al., 2017). Furthermore, the identification and characterization of helminth molecules and vesicles and the molecular pathways they target in the host represent the most valuable opportunity to develop tailored drugs inspired by nature that are efficacious, safe, and have minimal immunogenicity (Maizels and Gause, 2023; Maizels et al., 2018; Ryan et al., 2020; Drurey and Maizels, 2021), Figure 3.

The impressive molecular diversity of helminth excretory/secretory products, including a wide range of proteins and miRNAs, underscores their potential as therapeutic agents. While the development of recombinant expression systems for these molecules is crucial, challenges remain in optimizing production and delivery. The natural delivery of helminth miRNAs via EVs is a particularly intriguing strategy, and efforts to mimic this process using synthetic exosomes could revolutionize miRNA-based therapies (Ryan et al., 2020), see Figure 3.

The scientific community calls on the industry to make long-term investments in research aimed at deciphering and capitalizing on the extraordinary and diverse modes of action exhibited by these products. By unlocking the full potential of these natural compounds, we can pave the way for developing a new generation of innovative therapeutics.

## Conclusions

The ability of helminths to induce trained immunity offers a fascinating glimpse into the complex interplay between the immune system and parasites. By harnessing the power of these ancient organisms, researchers hope to develop innovative strategies for treating a range of inflammatory and autoimmune disorders. Identifying the specific regulatory pathways that underlie helminth-induced immune training is a major goal. By targeting these pathways, particularly during early childhood when the immune system is most malleable, scientists may be able to develop effective interventions, such as dietary, probiotic therapies, or even biological drugs to promote immune regulatory pathways and prevent the development of autoimmune diseases. ‘If we could find common pathways, we could adopt drugs or probiotics to activate [those pathways] to condition the immune system properly in early life,’ says Wills-Karp.
